# Fiber bundle shifting endomicroscopy for high-resolution imaging

**DOI:** 10.1364/BOE.9.004649

**Published:** 2018-09-06

**Authors:** Khushi Vyas, Michael Hughes, Bruno Gil Rosa, Guang-Zhong Yang

**Affiliations:** 1 Hamlyn Centre for Robotic Surgery, Imperial College London, South Kensington Campus, London SW7 2AZ, UK; 2 Applied Optics Group, School of Physical Sciences, University of Kent, Canterbury CT2 7NH, UK

**Keywords:** (170.2150) Endoscopic imaging, (110.0180) Microscopy, (100.3020) Image reconstruction-restoration

## Abstract

Flexible endomicroscopes commonly use coherent fiber bundles with high core densities to facilitate high-resolution *in vivo* imaging during endoscopic and minimally-invasive procedures. However, under-sampling due to the inter-core spacing limits the spatial resolution, making it difficult to resolve smaller cellular features. Here, we report a compact and rapid piezoelectric transducer (PZT) based bundle-shifting endomicroscopy system in which a super-resolution (SR) image is restored from multiple pixelation-limited images by computational means. A miniaturized PZT tube actuates the fiber bundle behind a GRIN micro-lens and a Delaunay triangulation based algorithm reconstructs an enhanced SR image. To enable real-time cellular-level imaging, imaging is performed using a line-scan confocal laser endomicroscope system with a raw frame rate of 120 fps, delivering up to 2 times spatial resolution improvement for a field of view of 350 *µ*m at a net frame rate of 30 fps. The resolution enhancement is confirmed using resolution phantoms and *ex vivo* fluorescence endomicroscopy imaging of human breast specimens is demonstrated.

## 1. Introduction

The ability to analyse tissue morphology in its natural biological environment, without excising a sample for pathology, could significantly impact the diagnosis and treatment of a range of diseases. Thin coherent waveguides such as optical fibers and fiber bundles have played a key role in bridging the gap between microscopes and endoscopes, providing a route to non-invasive cellular-level visualization and assessment of human tissue *in-vivo* and in real-time [1]. They have been used as compact endomicroscopic imaging probes for various high-resolution optical modalities, including epi-fluorescence microscopy [2,3], fluorescence confocal microscopy [4–6] optical coherence tomography [7,8] and two-photon microscopy [9].

Several configurations for fiber optic confocal endomicroscopes have been developed in the past. A detailed review can be found in [10]. They broadly fall into two categories: (i) distal scanning mechanisms using MEMS or piezo elements [6, 11, 12], and (ii) proximal scanning systems using bare fiber bundles or fiber bundles with distal optics [4, 5]. The former typically use a single optical fiber for light delivery and collection. This fiber is then itself scanned, or the exiting light falls on a micro-scanning element. This can achieve a good lateral resolution, comparable to a moderate-NA benchtop microscope. However, the typically large diameter of the distal tip (∼ 5 mm) and often low scanning rates (∼ 1 frame/second) greatly restricts their applicability for real-time clinical imaging applications.

Proximal scanning mechanisms, on the other hand, use a coherent fiber bundle instead of a single mode fiber as an image guide. By coupling a conventional external beam scanning unit or a camera to the proximal end of the fiber bundle, the size of the distal tip can be greatly reduced and the speed of image acquisition can be improved (typically 10 to 120 fps), thus making real-time *in vivo* imaging and video processing possible.

A significant problem with using fiber bundle endomicroscopes for point-of-care diagnostics, however, is the trade-off between the resolution and achievable field of view (FOV). This is because the quantity of fibers that can be packed into a single bundle (typically up to 30,000) limits the number of effective pixels of information in the image. Further, the inter-core spacing results in pronounced fiber-pixelation artefacts seen as a strong honeycomb pattern which reduces the contrast and spatial resolution of the image [5]. For such systems, the achievable resolution is not limited by diffraction or aberrations in the distal optics, but by the inter-core spacing, making it difficult to resolve sub-cellular features when imaging biological samples.

Previously reported fiber bundle endomicroscopy systems often utilized computational approaches such as Gaussian smoothing or linear interpolation between the cores to eliminate the pixelation artefacts [13–16]. However, these methods do not lead to an improvement in spatial resolution caused due to under-sampling. An alternate approach is to begin with a low resolution (LR) endomicroscopy image and attempt to increase the resolution by pixel Super-Resolution (p-SR) techniques. p-SR is a well explored topic in the machine vision community, utilizing sub-pixel source/image sensor shifting to create multiple under-sampled LR images and combining them to reconstruct a super resolution (SR) image [17]. For fiber bundle endomicroscopes, if the imaging probe shifts a small distance between the acquisition of two image frames, and this shift is not an integer multiple of the fiber core spacing along the direction of motion, then if the two images are registered and appropriately combined an enhancement in resolution can be obtained. Indeed, this tends to occur naturally during video mosaicking [18] or can be induced deliberately by causing random vibrations [19] or dithering [20, 21] of the fiber bundle. However, as these motions are essentially uncontrolled, any improvement in resolution is variable and may only occur along one direction. Further, for multi-frame methods, the enhancement is highly dependent on the performance of the registration algorithm, and hence on the signal-to-noise ratio of the images.

An alternative is to induce controlled micro-shifts of the fiber bundle by mechanical means. This concept was first suggested, although not demonstrated, for confocal endomicroscopy in [21, 22] and later shown in principle using a translation stage for epi-fluorescence endomicroscopy in [23, 24]. In [23], a fiber bundle was placed in direct contact with the sample and shifted laterally, using a bulky stage, in various patterns. Higher resolution images were then obtained by a reconstruction technique based on mean threshold bitmapping and a 2-fold resolution improvement was achieved. In [24], a maximum a posteriori (MAP) estimate of the HR image was calculated using conjugate gradient descent and a 2.8 times enhancement was achieved for imaging a USAF target using 16 LR images. Furthermore, an abstract from Cheng et al. suggested using a piezo tube for inducing the shift, and their work aimed at enhancing the resolution of two-photon endomicroscopy [25].

To date, these handful of studies have been initial proof-of-concept experiments only. Approaches relying on random motion may be too inconsistent for clinical use, while for induced motion, high-speed scanning systems are difficult to miniaturize and the system sacrifices image acquisition rate and probe size for improved image quality. Even if the current approaches were successfully miniaturized, when the moving fiber bundle is placed in direct contact with the tissue, friction and tissue deformation will tend to make them unreliable for clinical practice.

In this paper, a new high-resolution endomicroscope that incorporates a miniaturized piezoelectric tube scanner to induce precise micro-shifts of the fiber bundle is presented. To avoid problems with friction and tissue deformation, the fiber is scanned behind a miniature objective lens and does not make direct contact with the sample. Building on our preliminary work reported in [26], an optimal fiber-shifting pattern is derived and a fast Delaunay triangulation based p-SR algorithm is used to restore an SR image from multiple pixelation-limited LR images. The fiber shifting endomicroscope achieves a 2-fold improvement at 30 fps for a FOV of 350 *µm*. To validate the concept, a prototype fiber-shifting probe was assembled using a stock PZT tube and a GRIN lens and was employed in *ex vivo* tissue imaging studies.

## 2. Methods

### 2.1. Fiber-shifting endomicroscopy system

**Fig. 1:**
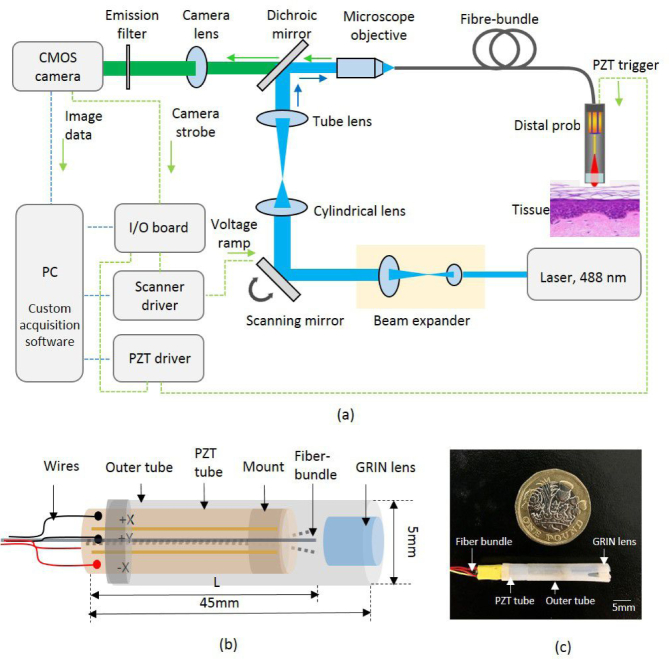
Schematics of (a) the line-scan confocal laser endomicroscopy (LS-CLE) system with (b) fiber-shifting distal probe. The proximal face of the fiber bundle is placed at the focal plane of the LS-CLE and the distal end is actuated by a PZT tube behind a GRIN lens with 1.92X magnification. (c) A photograph of the assembled 3D printed probe holder tube with 5 mm outer diameter. A UK one pound coin is shown for scale.

A schematic of the fiber-shifting super-resolution endomicroscopy system for fluorescence imaging is illustrated in Fig. 1(a)

. The proximal face of the fiber bundle is coupled to the optical imaging system which, for the purposes of this study, was a custom, high-speed, line-scan confocal laser endomicroscopy (LS-CLE) unit. The bundle was a fused Fujikura imaging fiber bundle (FIGH-30-850N) with approximately 30,000 cores. The Fujikura bundle chosen as this model is commonly used for endomicroscopy imaging due to the small core spacing and hence good intrinsic resolution. However, the proposed approach can be implemented using any endomicroscopy scanning system and fiber bundle without major modification.

A full description of the LS-CLE system’s operating principles can be found in [27]. In brief, a cylindrical lens (f=50 mm) is used to create a focused line from a 50 mW, 488 nm laser (Vortran Stradus, 488). A galvo-mirror (Thorlabs GVS001) sweeps the line across the proximal end of the fiber bundle in a direction perpendicular to the line. The bundle relays the line to the tissue *via* the distal GRIN objective, and returns collected fluorescence from all the points along the line, which is then imaged onto a monochrome rolling-shutter CMOS camera (Flea 3, FL3-U3-13S2M-CS). The rolling shutter of the CMOS camera operates as a virtual detector slit that rejects most of the out-of-focus light, leading to optical sectioning at frame rates of up to 120 Hz.

The optical system of the distal probe assembly is designed to achieve high magnification imaging in close proximity to the tissue surface. A schematic of the distal probe, which consists of the fiber bundle, a miniaturized quadruple PZT piezoelectric tube (PI Ceramics, PT230.94) and a high-NA (0.8) GRIN microlens is shown in Fig. 1(b). The lower end of the PZT tube is mounted in a custom 3D printed plastic holder while the upper end (tube tip) can freely move in all three dimensions. The fiber bundle is passed through the center of the PZT tube and rigidly secured to the distal end of the tube tip through a custom designed and 3D printed holder. For applications where the PZT is used for resonant scanning, the free-length of the fiber determines its resonance. However, here we make use of small displacements only, using the PZT for non-resonant scanning, and the free length of the fiber bundle was fixed to be 10 mm.

The outer surface of the PZT tube is separated into quartered electrodes. When two voltages equal in magnitude and opposite in sign are applied on two opposite quadrants, *x* or *y*, a bending motion is generated resulting in a lateral tip displacement along the respective axis. The inner surface of the tube is coated with an electrode, which is grounded in our experiments since we are interested only in transverse deflection. When the voltage *V_y_* is applied on the y-axis electrode pair, the deflection ∆*y* of a PZT tube can be expressed by the following equation:

$$\Delta y=\frac{2\sqrt{2}d_{31}V_{y}L^{2}}{\pi (D+h)h}$$ (1)

where *d*_31_ is the piezoelectric strain constant, *V_y_* is the applied voltage, *L* is the tube length, *D* is the inner tube diameter and *h* is the thickness of the tube. For tube diameter much greater than wall thickness, (*D* + *h* ) ≈ *D* and Eq. (1) leads to Chen’s result for tube deflection [28].

A stock 1.4 mm diameter gradient index (GRIN) micro-lens assembly (GRINTech GT-MO-080-0415-488) is fixed in front of the fiber bundle such that the distal tip of the fiber is imaged onto a plane approximately 80 *µ*m deep in the tissue, with a 1.92X magnification factor. As the GRIN lens does not move, this avoids friction between the moving fiber and the tissue which would otherwise make the scanning less reproducible due to tissue deformation. The entire probe assembly is encased in a custom 3D printed plastic tube with a 45 mm rigid length (including the GRIN lens) and 5 mm outer diameter (OD). A photograph of the prototype probe is shown in Fig. 1(c).

In standard-resolution operation, the camera is operated in free-run mode, which allows low-resolution (LR) images to be acquired at the full frame rate of 120 fps, by generating a trigger pulse on its strobe output pin at the start of each frame acquisition. The pulse triggers the analog output of a data acquisition card (NI-USB 6211) which has a 16-bit, 250 KS/s sampling rate, to send a ramp voltage signal to the galvo-scanning mirror, with some user-specified delay. In super-resolution mode, activated by the click of a button on the custom control software, the trigger pulse also triggers the delivery of a series of drive signals to the PZT tube, as explained in the next section. This results in the fiber bundle being shifted to a series of different positions, synchronised such that one image frame is acquired for each position. An experimentally determined delay is provided such that the data acquisition starts once the fiber bundle reaches each stationary position, and an image frame is acquired for each position of the PZT tube at 120 fps.

### 2.2. Electronic circuit for PZT actuation

**Fig. 2:**
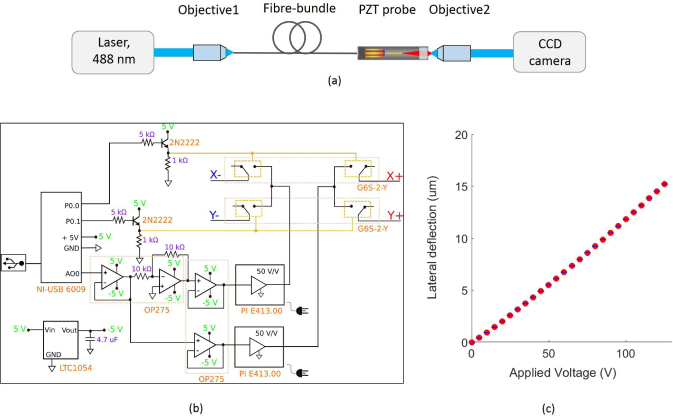
(a) PZT characterization test: Optical setup for illuminating a single core of the fiber bundle and estimating the tip deflection by tracking the position of the centroid of the focused spot on the CCD camera. Objective1 is microscope objective with 40X magnification and Objective2 is with 20X magnification. (b) Schematics of PZT driver circuit. (c) 2-D plot of fiber bundle deflection *versus* applied voltage on the PZT electrode pair.

A data acquisition card (NI-USB 6009) was used to produce a DC voltage, in the range from 0 V to 5 V, from one of the analogue outputs of the card (AO0), as depicted in Fig. 2(b)

. This voltage was split into two different signals with equal magnitude and opposite sign through an inverting amplifier (Analog Devices, OP275) with unitary gain, followed by signal buffering *via* two voltage-followers (Analog Devices, OP275), before being fed as the input for two PZT amplifiers (PI, E413.00) with gain of 50 V/V and output span between −250 V and 250 V and connected to the power grid line. The routing of these amplified signals or drive voltages to the corresponding PZT electrode pair was achieved by two solid state relays (Omron, G6S-2-Y) that, when triggered, excite the electrodes independently. The trigger signals were generated by two digital outputs from the acquisition card with 5 V of magnitude. Since the electronic current provided by the card was not enough to activate the relays, due to the low impedance of their coils, an additional transistor (Multicomp, 2N2222) in a common-collector configuration was employed to supply a larger current to the relays and activate the switching mechanism. A charge pump device (Linear Technology, LTC1054) was used to produce a negative 5 V supply for all the electronic components except the PZT amplifiers, as the acquisition card could only generate the positive 5 V. Symmetric supply levels were required to accommodate the bipolar signals leading to the PZT amplifiers.

### 2.3. PZT characterization

Following probe assembly, the dependence of the fiber bundle tip deflection on the voltage applied to the PZT electrodes was measured. To achieve this, the distal end of the fiber bundle was imaged onto a camera using a microscope objective using the experimental set-up shown in Fig. 2(a). The output of a laser diode (Vortran, 488) was focused onto the fiber bundle *via* Objective1 with 40X magnification. The transmitted light was imaged onto a monochrome CCD camera (Thorlabs, DCU224C, 1280 × 1024 pixels) *via* a 20X microscope Objective2. When a single core of the fiber bundle was illuminated, the output showed some distribution of power among neighboring cores, indicating that inter-core coupling was occurring. An intensity thresholding algorithm was applied to eliminate the low-intensity neighboring pixels and a centroid estimation algorithm was used to find the center of a single core of the fiber bundle.

Input voltages in the range of 0 to 125 V with a step-size of 5 V were provided to the *x* and *y* axis of the PZT tube scanner from the PZT amplifiers. For each axis, the lateral displacement of the fiber bundle tip was estimated by tracking the position of the centroid of the focused spot on the CCD camera, averaged over 5 runs. A representative plot of deflection versus applied voltage along the x-axis is shown in Fig. 2(c). As the input voltage is increased, the displacement of the fiber bundle tip gradually increases. From these direct measurements, a voltage shift of 5 V was determined to correspond to a tip deflection of (0.61 ± 0.03) *µ*m. For the Fujikura fiber bundle with an inter-core spacing of 4.48 *µ*m (estimated from an SEM image of the fiber bundle), the desired shift is half the core spacing, i.e. about 2.24 *µ*m corresponding to a drive voltage of 19 V.

Using the theoretical model given in Eq. (1) for the stock PZT tube from PI Ceramics (PT230.94) with quoted dimensions 30(L) × 3.2(OD) × 2.2(ID) mm, *d*_31_ of −180 × 10^−12^m/V, a drive voltage of 19 V should result in a lateral deflection of about 2.05 *µ*m. By assuming that the fiber bundle inside the PZT tube, with fiber free-length of 10 mm, will experience lateral deflections proportional to its length (considering only deformations in the elastic regime and without torsion occurring along the fiber length), the estimated deflection of the fiber tip is about 2.73 *µ*m. In practice we measured a smaller deflection which we attribute to the stiffness of the fiber bundle.

### 2.4. Super resolution (SR) image reconstruction

**Fig. 3:**
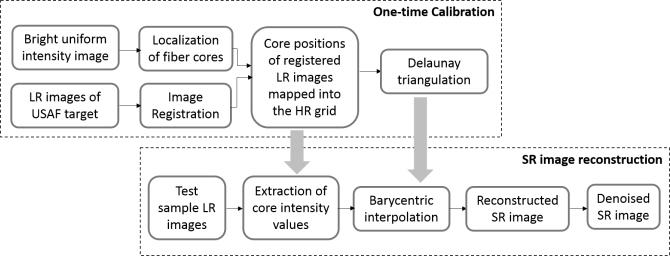
Sequence of steps illustrating Delaunay triangulation based reconstruction of SR image from a set of fiber-bundle pixelation limited LR image frames

The SR image reconstruction task is divided into two stages: a one-time calibration and then subsequent reconstruction of each SR image as depicted in Fig. 3

. The calibration stage involves identifying the position of the center of each core in the bundle, and determining the geometric transformation matrix for each step of the fiber bundle shifting pattern driven by the PZT. In the second stage, a fast Delaunay triangulation (DT) based interpolation algorithm is used to reconstruct a SR image from the multiple LR images acquired at each position. The approach is similar to that proposed by [29], adapted to improve resolution of fiber bundle endomicroscopy images.

Prior to acquisition, a dark background calibration is performed by recording 50 frames with the tip of the probe covered. The probe is then pointed at a bright uniform target, and the core-center positions are detected using a Hough transform. A circular area of interest is taken using a convex hull algorithm to remove artefacts from the edges of the fiber bundle, leading to final image diameter of 350 *µm*.

The probe is then pointed at an object with high-resolution detail (such as a USAF resolution target). The chosen pattern of fiber bundle shifts is run, LR images are acquired from each shifted position and the background image is subtracted from each. A sub-pixel frequency domain based phase correlation technique presented in [30] is then used to estimate the geometric transformation between the consecutive LR image frames. The geometric transformation is made in one step using a discrete Fourier transform, thus making it high-speed. Given two LR images, *f*_1_(*x, y*) and *f*_2_(*x, y*), shifted horizontally and vertically by (Δ*x*, Δ*y*) with respect to each other, in the Fourier domain their relationship can be expressed as:

$$F_{2}(u,v)=e^{2\pi i(u^{T}\Delta x+v^{T}\Delta y)}F_{1}(u,v)$$ (2)

where *F*_1_(*u, v*) and *F*_2_(*u, v*) are 2D Fourier transforms of *f*_1_(*x, y*) and *f*_2_(*x, y*) respectively. For N image frames, the shift parameters (Δ*x*) and (Δ*y*) between every image frame *f_k_*(*x, y*) and the first image *f*_1_(*x, y*) are computed from Eq. (2) as the least-squares solution of the slope of the phase difference.

An array of core-center positions is then assembled from the measured core positions for each position of the fiber bundle, with each set of core positions shifted using the estimated shift parameters (Δ*x_k_*, Δ*y_k_*). If there are *N* cores in the bundle, and *p* fiber bundle positions are used, this results in a total of *Np* core positions. A Delaunay triangulation (DT) is then formed over these *Np* core positions by first computing the Voronoi diagram. The Voronoi diagram decomposes the HR reconstruction grid into regions around each core position such that all the points in the region around each core, *c_i_*, are closer to *c_i_* than any other core. A Delaunay triangulation mesh is constructed by connecting points with which the Voronoi cells have common boundaries such that every pixel is enclosed in one triangle with vertices corresponding to the closest three core-center positions. A reconstruction grid is chosen, the enclosing triangle is identified for each pixel, and each pixel location is converted to triangular barycentric co-ordinates (a measure of its distance from each vertex of the enclosing triangle). For cores *c_i_* at vertex *i*, for *i* = 1, 2, 3, *p* as the pixel location and *A*_1_, *A*_2_ and *A*_3_ to be the area of three triangles *c*_2_*c*_3_*p*, *c*_1_*c*_3_*p* and *c*_1_*c*_2_*p*, the barycentric co-ordinate *b_i_* is calculated as:

$$b_{i}=\frac{A_{i}}{A_{1}+A_{2}+A_{3}}\;where\;i=1,2,3$$ (3)

This concludes the one-time calibration, which is used as the input to the reconstruction of all subsequent SR images.

During imaging, following acquisition of the set of LR images from each shifted position, the core intensity is extracted from each core in each image. The resulting SR image is reconstructed by assigning each pixel an intensity value, *I_p_*, obtained by triangular linear interpolation between the intensity values of the three nearest cores of the enclosing Delaunay triangle using:

$$I_{p}=b_{1}I_{C,1}+b_{2}I_{C,2}+b_{3}I_{C,3}$$ (4)

where *I_C,i_* is the intensity values of the core at vertex *i* and *b_i_* is the corresponding pre-calculated barycentric co-ordinate for that reconstruction pixel *p*. In the last step, a median filter is used to remove salt-and-pepper impulse noise from the reconstructed SR image. For the protoype system reported here, LR images were recorded at 120 fps and then processed offline, but the reconstruction step is computational inexpensive and could be implemented in real-time.

## 3. Results

### 3.1. Evaluation of scanning patterns

The distribution of energy within each fiber core, and hence the area of the sample that each core integrates over, follows an approximately Gaussian profile, with a mode field diameter (MFD) smaller than the spacing between adjacent cores. Knowledge of the core-spacing and MFD is therefore necessary to determine the scanning pattern with the optimal number and magnitude of fiber shifts in order to enhance resolution while maintaining an acceptable frame rate. For the fiber bundle, the core diameter and inter-core spacing were estimated by acquiring SEM images as 2.45 *µ*m and 4.48 *µ*m respectively. The MFD was experimentally measured (by imaging onto a camera with 20X microscope objective) to be about (4.23 ± 0.14) *µ*m, and the full width half maximum (FWHM) to be (2.49 ± 0.08) *µ*m.

Applying the Rayleigh criterion for an Airy disc with the same FWHM (i.e. with a first minimum at 1.19 times the FWHM), the minimum fiber bundle shift necessary to obtain two resolved peaks would be approximately 2.96 *µ*m. Using the Sparrow criterion the required shift is about 2.12 *µ*m. If we compare these values to the core spacing of 4.48 *µ*m, it is clear that there is significant under-sampling occurring in conventional fiber bundle systems, and a potential for up to a 2-fold resolution improvement by fiber shifting.

**Fig. 4:**
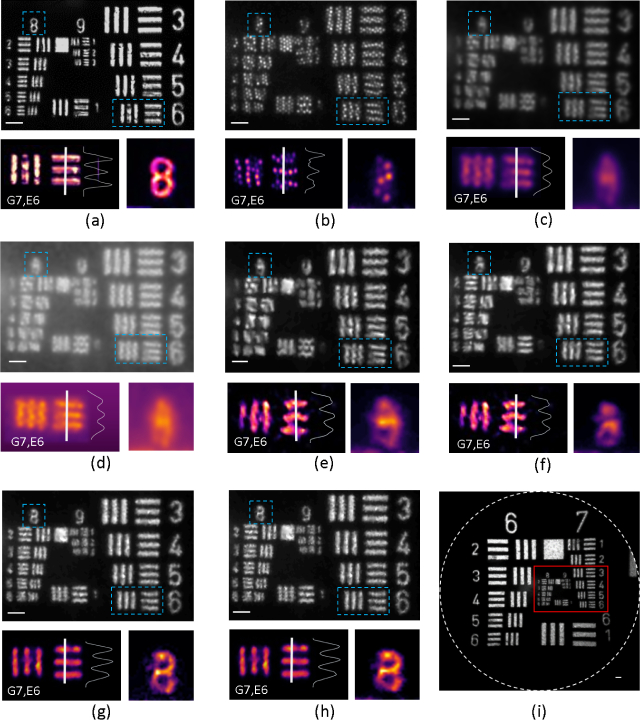
Cropped images of USAF resolution target back-illuminated by a green LED, with fiber shifting motion performed using a translation stage. Large images show Group (G) 7, Elements (E) 3–6, and all elements of Group 8 and 9. Smaller zoomed images show G7,E6, a 2-D plot of the intensities of pixels along the line segment shown by a white line on G7,E6, and the numerical ‘8’. (a) Image acquired with the LS-CLE system and 1.92X GRIN lens (no fiber bundle). (b) Raw experimental LR input image acquired with a fiber bundle and 1.92X GRIN lens. (c–e) Image reconstructed by (c) Gaussian smoothing (*σ* = 1.7 pixels), (d) Gaussian smoothing with pre histogram equalization and (e) DT algorithm on a single LR image. The respective SR images reconstructed using the proposed method are shown for (f) a 1-D shift pattern where 2 images are acquired with shift of 2.24 *µ*m, (g) a 2-D shift pattern where 4 images are acquired in a square pattern with a shift of 2.24 *µ*m, and (h) a 2-D shift pattern where 8 images are acquired with a 1.12 *µ*m inter-image shift. (i) Single un-cropped image representing all elements of groups 6–9 of USAF target reconstructed using the DT algorithm and 2×2 pattern. Full field of view of each acquired image (in white circle) is 350 *µ*m. Region of interest (marked in red) corresponds to image (g). The scale bar is 10 *µ*m.

To determine the desired scanning pattern, independently from the performance of the PZT, we tested the use of 1-D linear and 2-D square scanning patterns using a motorized translation stage (8MT173, Standa Ltd.). A simple test involved imaging a high-resolution 1951 USAF target consisting of 9 groups of horizontal and vertical line pairs with various spacings. As the target was not fluorescent, it was back-illuminated by a green LED and imaged in transmission. Figure 4(a)

shows the image acquired with the LS-CLE system and 1.92X GRIN lens (no fiber bundle). This represents the fundamental limit on resolution from diffraction and aberrations in the optics.

Figure 4(b) shows an image of the target through the fiber bundle and 1.92X GRIN micro-lens without any processing. This and subsequent images are cropped from the full field of view which is 350 *µm*. Figure 4(c) shows an image reconstructed by the Gaussian smoothing (*σ* = 1.7 pixels) and Fig. 4(d) by Gaussian smoothing with a pre-histogram equalization as proposed in [14] on a single LR image. Figure 4(e) shows an image reconstructed by the DT algorithm on a single LR image. Figures 4(f) and (g) show the results of applying the proposed SR technique using 1-D and 2-D square patterns with the fiber bundle shifted by half the inter-core spacing between images. This corresponds to combining 2 images in Fig. 4(f) and 4 images in Fig. 4(g) with 2.24 *µm* inter-image shift. For better visualization, a cropped image of high resolution features consisting of Group 7, elements 3–6, and all elements of Group 8 and 9 are presented. The zoomed insets correspond to Group 7, element 6 (G7,E6) and the numeral ‘8’.

The Nyquist frequency of the bare fiber bundle corresponds to approximately 112 lp/mm. Due to the 1.92X magnification of the GRIN microlens, the Nyquist frequency of the fiber bundle with lens corresponds to approximately 215 lp/mm. The smallest line pairs on the USAF target that can be completely resolved for a single LR image are of Group 7, Element 6, as shown in Fig. 4(b). This corresponds to 228.1 lp/mm and a bar width of 2.19 *µ*m. For the image reconstructed by applying only Gaussian smoothing, Gaussian smoothing with a pre histogram equalization and the DT algorithm to a single LR image, the pixelation artefacts are reduced, due to which line pairs from Group 8, Element 1, with a spatial frequency of 256 lp/mm can be resolved. However, no significant improvements in spatial resolution can be observed. For the reconstructed image using a 1-D shift only, although the image quality is enhanced, the resolution enhancement is somewhat directionally dependent. The smallest resolvable line pairs correspond to Group 8, Element 2, a spatial frequency of 287.4 lp/mm as shown in Fig. 4(f). When LR images which are shifted in a 2D square pattern are combined using the proposed SR algorithm, the smallest resolvable lines are Group 8, element 6, as shown in Fig. 4(g). This corresponds to a spatial frequency of 456.1 lp/mm and a bar width of 1.1 *µ*m, resulting in an approximately 1.8X resolution improvement compared with reconstruction from a single image.

We then compared the spatial resolution improvement from the 2D square pattern when the fiber bundle is shifted by half and one-fourth of the inter-core spacing, corresponding to 4 images with 2.24 *µ*m and 8 images with 1.12 *µ*m inter-image shift, shown in Figs. 4(g) and (h) respectively. It is observed that the 2-D square pattern provides about 2-fold resolution improvement whether 4 or 8 images are used, broadly as expected from the measurement of the core spot size. Using the LS-CLE system with an image acquisition rate of 120 fps, for an SR image reconstructed from 4 LR images, an overall acquisition rate of 30 fps can be achieved which makes it suitable for real-time imaging. Given the significant frame rate penalty of using 8 images without any noticeable further resolution improvement, the 2×2 pattern was selected for use with the prototype probe as the optimal fiber shifting pattern for biological tissue imaging experiments. Figure 4(i) shows an un-cropped image of all elements groups 6–9 of USAF target reconstructed using the DT algorithm and 2×2 pattern for reference.

### 3.2. Probe spatial resolution estimation

**Fig. 5:**
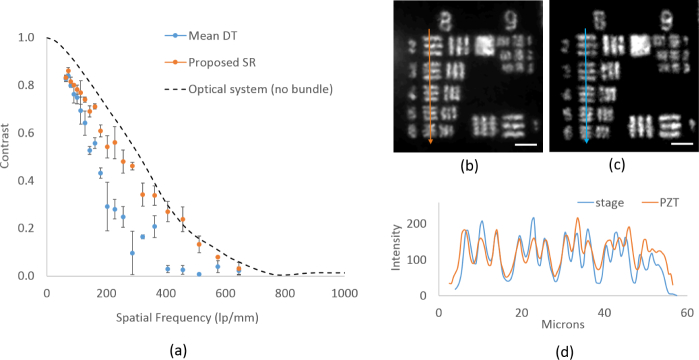
(a) Square wave modulation contrast obtained by applying the DT algorithm on the average of 4 frames and the proposed SR method. This is compared with imaging through the 1.92X GRIN lens optical system with no imaging bundle. Image of USAF target showing all elements of Groups 8 and 9, reconstructed using the proposed method where fiber shifts are generated using (b) PZT scanner and (c) motorized translation stage. (d) Shows 2-D graph of the intensities of pixels along a line segment on G8, E2-6. Scale bar is 10 *µ*m.

To determine the spatial resolution of the prototype probe, the square wave transfer function, which is a similar concept to the modulation transfer function, was determined by finding the observed modulation depth across all elements of USAF target Groups 6–9, averaged over 3 runs. Four image frames were acquired by scanning the imaging probe in a 2-D square pattern, with the fiber bundle shifted by half the inter-core spacing, and the contrast was measured for the SR image reconstructed using the algorithm described above. We compared this with the contrast of an image reconstructed using the same DT algorithm applied to a simple average of four frames and to an image acquired directly through the 1.92X GRIN lens with no fiber bundle. The observed modulation depth (square wave contrast) of the USAF bar patterns is plotted against their spatial frequencies in Fig. 5(a)

.

We first consider the contrast of Group 7, Element 6 on USAF target (228.1 lp/mm) as it is closest to the Nyquist frequency of the fiber bundle with the GRIN lens. The average modulation depth was calculated as 55.9% using the proposed method, improved from 28% for the average of four LR images. Qualitatively, the PZT based fiber-shifting probe can resolve Group 8, Element 6 of the USAF target, which corresponds to 456.1 lp/mm or a bar width of 1.1 *µ*m, as shown in Fig. 5(b). The measured contrast using the proposed SR algorithm at 456.1 lp/mm was 29.7% while that for the averaged LR images was 2.6%. Figure 5(c) shows an image of Group 8 and 9 of USAF target obtained when a programmable translation stage was used for fiber-shifting. By comparing the pixel intensities along a line-segment on G8, E2-6 of Figs. 5(b) and (c) it can be seen that the resolution enhancement obtained using the PZT based prototype probe is comparable to that obtained using the translation stage, as shown Fig. 5(d).

### 3.3. Imaging results

The performance of the fiber shifting endomicroscopy system was tested by imaging lens tissue cleaning paper and *ex vivo* human breast tissue. In standard resolution mode, images were acquired at 120 fps and in SR mode four images were acquired in a 2-D square pattern at 30 fps. The deflection voltage applied to each electrode-pair of the PZT tube was ± 19V, corresponding to a 2.24 *µ*m shift. The one-time calibration step to determine the core-center positions and shift parameters was performed by repeating the 2D square scanning pattern multiple times on a USAF resolution target. For each axis, the standard deviation of the shift, averaged over 5 runs was about 0.12 *µ*m. The estimated shift values were then used as the input for SR image reconstruction for all the test samples. All processing was performed offline in MATLAB, although the system is suitable for real-time applications.

Figure 6

**Fig. 6:**
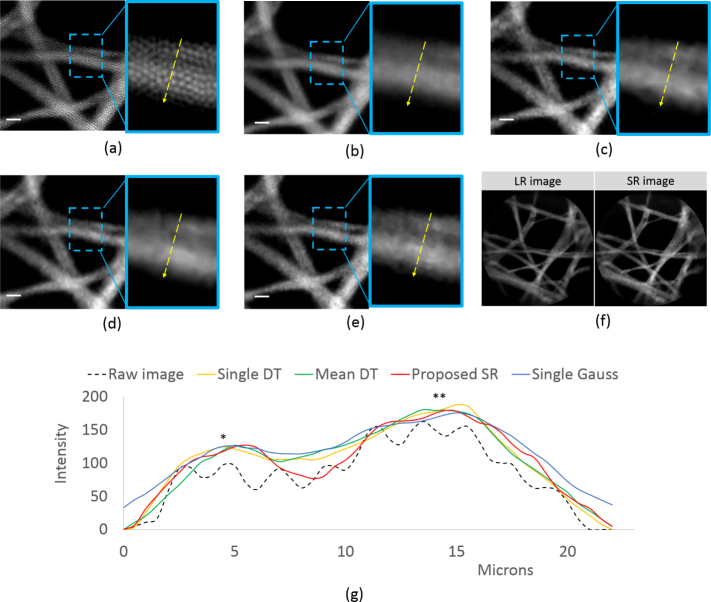
Results from imaging lens tissue paper using four images with a 2×2 square shift pattern, showing (a) single raw acquired LR image, (b) reconstruction by Gaussian smoothing on a single LR image, (c) reconstruction by DT algorithm on a single LR image, (d) reconstruction by DT algorithm on the average of 4 shifted LR images, (e) reconstruction using the proposed SR method and (f) un-cropped images of single LR frame and SR image reconstructed using the proposed method. For (a)–(e), images are cropped to 233×233 pixels for better visualization. Zoomed insets (3.1X magnification) correspond to a small area where two lens paper fibers overlap. (g) Plot of pixel intensity along a line segment shown on the insets. Image contrast values are calculated at *peak-1 and **peak-2. The scale bar is 10 *µ*m.

shows cropped images, with zooms in the insets, of lens tissue paper stained with 0.02% acriflavine hydrochloride solution. Four image frames were acquired by scanning the imaging probe in the 2-D square pattern with 2.24*µ*m inter-image shifts. For comparison, a single acquired LR image, labeled as ‘Raw Image’, is shown in Fig. 6(a), as well as the Gaussian smoothing (*σ* = 1.7 pixels) and the DT algorithm reconstruction of this single LR raw image, labeled ‘Single Gauss’ and ‘Single DT’ in Figs. 6(b) and (c) respectively. An image reconstructed using the DT algorithm on an average of four acquired frames, labeled ‘Mean DT’, is shown in Fig. 6(d), and an image reconstructed using the proposed SR algorithm, labeled ‘Proposed SR’, is in Fig. 6(e). Full field of view images of a single LR frame and SR image reconstructed using the PZT based fiber-shifting probe and proposed SR algorithm are shown in Fig. 6(f).

For Figs. 6(a)–(e), a small area where two lens paper fibers overlap was chosen and magnified 3.1 times for visualization purposes. The intensity values along a yellow line are plotted in Fig. 6(g). For the raw image, the fiber pixelation artefacts lead to significant intensity modulations making it difficult to distinguish fiber strands of the lens tissue paper. For the ‘Single Gauss’, Single DT’ and ‘Mean DT’ reconstruction, although the fiber-cores are no longer visible, the edges appear fuzzy and image contrast at the peaks corresponding to the center of each lens paper fiber is low: 5%, 8.2% and 10.8% for peak-1 and 21.3%, 28.0% and 27.8% for peak-2 respectively. Using the proposed SR algorithm the two fibers of lens tissue paper are clearly distinguishable, resulting in narrower and well-defined peaks with image contrast values of 24.6% for peak-1 and 40.0% for peak-2.

**Fig. 7:**
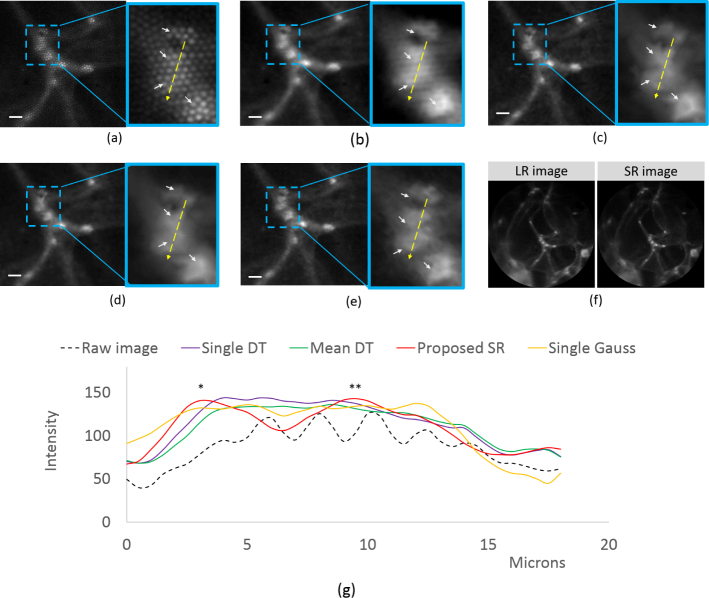
Results from imaging adipose cells of human breast tissue using four images with a 2×2 square shift pattern, showing (a) raw single acquired LR image, (b) reconstruction by Gaussian smoothing on a single LR image, (c) reconstruction by DT algorithm on a single LR image, (d) reconstruction by DT algorithm on the average of 4 images, (e) reconstruction using the proposed SR method and (f) un-cropped images of single LR frame and SR image reconstructed using the proposed method, with field of view of 350 *µ*m. For (a)–(e), images are cropped to 233×233 pixels for better visualization. Zoomed insets (3.1X magnification) correspond to a small area containing adjacent nuclei on the borders of adipose cells.(g) Plot of pixel intensity along a line shown in the insets. Image contrast is calculated at *peak-1 and **peak-2. The scale bar is 10 *µ*m.

We then performed fluorescence fiber bundle endomicroscopy imaging of normal adipose cells of human breast tissue. Small cut-outs (2 mm × 2 mm) were sectioned from the tissue specimen and stained using acriflavine hydrochloride 0.02% in saline solution. The specimen was immersed in a test tube containing the staining solution for 1 minute and then rinsed with water to remove excess stain, before being imaged immediately. Figure 7(a)–(e)

shows cropped images and zoomed insets of stained adipose cells of normal breast tissue. Figure 7(f) shows un-cropped images of a single frame and reconstruction using the proposed SR algorithm. The adipose cells appear as dark hexagons with bright borders. There are sparse nuclei on the borders which are positively stained by the dye and can be clearly distinguished as hyper-fluorescent dots [31]. LR and SR images were reconstructed as for the tissue paper.

For Figs. 7(a)–(e), a small area where two nuclei are close to each other was chosen and magnified 3.1 times, as shown in the insets, for visualization purposes. The intensity values along a yellow line are plotted as a function of distance in Fig. 7(g). From the intensity plot, it is evident that for the raw image, the fiber-pixelation artefacts lead to significant intensity modulations making it difficult to identify any underlying structures. For ‘Single Gauss’, ‘Single DT’ and ‘Mean DT’, the profile appears as a single broad band with some modulations in intensity but the contrast for them is significantly low, less than 1.3%. As a result, the two nuclei cannot be resolved. Using the proposed SR algorithm, two peaks corresponding to the two nuclei are observed with image contrast values of 14.0% for peak-1 and 12.2% for peak-2, making it possible to resolve the two neighboring nuclei, which otherwise was not possible.

## 4. Discussion

The imaging results demonstrate that the proposed PZT based fiber shifting system allows for an enhancement of the resolution compared to reconstructions based on single images. These experiments were conducted using a line-scan confocal laser endomicroscopy system because of the high image acquisition rate of 120 fps. With the proposed system, SR images can be acquired at 30 fps, making it suitable for real-time imaging applications. In principle, the proposed system can be implemented with any microscope and fiber bundle without major hardware modification. The reconstructed images demonstrate higher contrast, and details such as nuclear shape are more readily visualized in the zoomed-in sections.

In the literature, several nuclear morphometric metrics such size, shape and number in a given area, as well as nucleus/cytoplasm ratio, have been shown to help distinguish between normal, benign and neoplastic breast conditions, making it important to resolve each nuclei accurately [32]. The preliminary experiments reported here demonstrate that the system has sufficient resolution to resolve features separated by less than 2.2 *µ*m (on the USAF target) and nuclei with a diameter of about 2.5 *µ*m. Considering that the neoplastic tissue exhibits an increase in size for the population of nuclei, these *ex vivo* imaging results suggest a potential benefit of this system for cancer diagnosis by real-time assessment of epithelial structures with sub-cellular resolution. However, further work will be required to determine the applicability of this work to nucleic imaging more generally, and to establish whether there is a significant benefit over lower-resolution approaches.

The prototype probe was constructed using a stock GRIN lens and PZT tube. A drive voltage of 19 V was applied to each electrode-pair of the PZT tube to achieve the required lateral deflection of 2.24 *µ*m, which is equal to half the inter-core spacing of the Fujikura fiber bundle. This is below the stipulated limit of 42.4 V peak AC as per the IEC 60601-1 standard, making the approach suitable for clinical *in vivo* imaging.

The entire probe assembly had a 45 mm rigid length (including the GRIN lens) and a 5 mm outer diameter (OD). This makes the device currently too large to be used through most endoscope working channels. The limiting elements of the design are the PZT tube, with dimensions 30(L) × 3.2(OD) × 2.2(ID) mm, and the 3D printed outer tube. Future designs could use custom-made smaller PZT scanning tubes for better compactness, as well as thinner-walled outer packaging. Such a system could be deployed through the working channel of conventional endoscopes and provide the basis for improving the diagnostic performance of optical biopsy systems, increasing the ability to identify and differentiate features of normal and neoplastic cells at sub-cellular scale.

As a multi-frame techniques, the approach requires minimal motion between image frames in order to function correctly. Hence, the method reported here requires that the probe is held steady against the tissue, and could not be used with video mosaicking techniques. When motion is present, it would be possible to adapt the algorithm to instead make use of the motion of the probe, rather than the controlled motion of the PZTs, for super-resolution. However, at this point, the repeatability benefits of the approach would be lost.

The two-fold improvement in resolution was achieved using a Delaunay triangulation based SR construction algorithm and a 2×2 scanning pattern. Using this algorithm, no benefit was found to the use of a more dense scanning pattern. However, it is possible that a further resolution enhancement could be obtained by using different reconstruction algorithms and scanning patterns. A large number of pixel-super-resolution algorithms have been developed for other applications, with a comparison of the performance and computation time of some such approaches available in [24,33]. It may be possible to adapt these algorithms to this application and develop customized scanning patterns to exceed the gains demonstrated here.

## 5. Conclusion

We have developed a miniaturized, high-speed PZT-based fiber shifting endomicroscope to enhance the resolution over conventional fiber bundle based imaging systems. The fiber shifting endomicroscope provides almost a two-fold improvement in resolution, and coupled to a high-speed scanning system could provide real-time imaging of biological samples at 30 fps. The approach can be used for other fiber bundle based imaging systems, providing that a four-fold reduction in net frame rate is acceptable. By improving the resolution while maintaining a large field-of-view, this technique could potentially provide the basis for improving the diagnostic abilities of endomicroscopes in the clinic.
